# Supplementary material for: Links between meaning in life and physical quality of life after rehabilitation: Mediating effects of positive experiences with physical exercises and mobility
Source: PLoS One. 2019 Oct 31;14(10):e0224503. doi: 10.1371/journal.pone.0224503 (PMC6822941; doi:10.1371/journal.pone.0224503)
Supplement: S1 Table — Abbreviations: ICD 10: The International Statistical Classification of Diseases and Related Health Problems, 10th revision (WHO, 2016), M: mean, SD: standard deviation. (DOCX) [file pone.0224503.s001.docx]

| Group | *n* | Time since diagnosis (in months) | | Subgroup | ICD-10 diagnosis type | % |
| --- | --- | --- | --- | --- | --- | --- |
|  |  | *M (SD)* | *Range* |  |  |  |
| Central nervous system diseases | 89 | 81.37 (89.62) | 1-600 | Cerebrovascular diseases, e.g. disorders of brain | G.00 - G.99, except for  G.30-37 | 65.1 |
|  |  |  |  | Demyelinating diseases of the central nervous system, e.g. multiple sclerosis | G.30 - G.37 | 18.6 |
|  |  |  |  | Other diseases of the central nervous system, e.g. in situ neoplasms | D.00 - D.09 | 16.3 |
| Musculoskeletal diseases | 250 | 85.65 (92.88) | 1-526 | Dorsopathies, e.g. discopathy | M.40 - M.54 | 48.8 |
|  |  |  |  | Arthropathies, e.g. intra-articular knee lesions | M.00 – M.25 | 9.6 |
|  |  |  |  | Injuries to the shoulder and upper arm, injuries to the elbow and forearm, injuries to the wrist and hand, e.g. injury of shoulder and upper arm | S.40 - 69 | 8.0 |
|  |  |  |  | Injuries to the hip and thigh, injuries to the knee and lower leg, injuries to the ankle and foot, e.g. injury of knee and lower leg | S.70 - 99 | 13.6 |
|  |  |  |  | Diseases of the musculoskeletal system and connective tissue and congenital malformations, deformations and chromosomal abnormalities but no dorsopathies and arthropathies, e.g. other and unspecified osteoarthritis | M.00 - M.99 and Q.00 - Q.99, except for  M.00 - M.25, M.40 - M.54 | 12.4 |
|  |  |  |  | Other musculoskeletal diseases, e.g. injuries to the neck | S.10 - S.19 | 7.6 |
