# Supplementary material for: Links between meaning in life and physical quality of life after rehabilitation: Mediating effects of positive experiences with physical exercises and mobility
Source: PLoS One. 2019 Oct 31;14(10):e0224503. doi: 10.1371/journal.pone.0224503 (PMC6822941; doi:10.1371/journal.pone.0224503)
Supplement: S2 Table — All variables were measured at Time 1. Abbreviations: M: mean; SD: standard deviation; a p-values in bold indicate statistical significance; b economic status was measured by a 3-point scale: 1—the economic situation worse than the average family in Poland, 2—similar to the economic situation of an average family in the country, 3 –better; c education was measured by a 3-point scale: 1—primary education or vocational education (no high school education), 2—secondary education, 3—higher education; d employment was measured by a 2-point scale: 1—employed (full- or part-time), 2—unemployed (or being retired or a pensioner); b, c, d for percentage of categories for economic status, education level, and employment see Table 1 (the manuscript). (DOCX) [file pone.0224503.s002.docx]

| Scale | Completers | | Non-completers | |  |  |  |
| --- | --- | --- | --- | --- | --- | --- | --- |
|  | *M / %* | *SD* | *M / %* | *SD* | *F or χ^2^* (*df*) | *p* | η² |
| Meaning in life | 3.74 | 0.92 | 3.88 | 0.91 | 1.80 (1, 337) | .18 | .005 |
| Physical quality of life | 2.95 | 0.53 | 2.92 | 0.46 | 0.01 (1, 337) | .66 | .001 |
| Positive experiences with physical exercises | 3.08 | 0.73 | 3.01 | 0.65 | 0.88 (1, 337) | .34 | .003 |
| Mobility | 3.36 | 0.88 | 3.56 | 0.66 | 4.04 (1, 337) | **.04^a^** | .012 |
| Age | 54.83 | 12.03 | 53.46 | 0.69 | 1.07 (1, 337) | .30 | .003 |
| Time since diagnosis (months) | 85.88 | 94.76 | 81.51 | 85.62 | 0.16 (1, 337) | .68 | .000 |
| Economic status^b^ | 3.05 | 0.91 | 2.97 | 0.89 | 0.65 (1, 337) | .42 | .002 |
| Education level^c^ | 3.16 | 1.30 | 2.92 | 1.12 | 2.66 (1, 337) | .10 | .008 |
| Employment status^d^ (% employed) | 57.6% |  | 42.4% |  | 30.71 (2, 339) | **<.001^a^** |  |
| Female gender | 71.1% |  | 28.9% |  | 0.91 (2, 339) | .33 |  |
| Type of diagnosis |  |  |  |  | 33.14 (2, 339) | **<.001^a^** |  |
| CNSD | 93.3% |  | 6.7% |  |  |  |  |
| MSD | 60.4% |  | 39.6% |  |  |  |  |
