# Supplementary material for: Links between meaning in life and physical quality of life after rehabilitation: Mediating effects of positive experiences with physical exercises and mobility
Source: PLoS One. 2019 Oct 31;14(10):e0224503. doi: 10.1371/journal.pone.0224503 (PMC6822941; doi:10.1371/journal.pone.0224503)
Supplement: S3 Table — a p-values in bold indicate statistical significance. Abbreviations: QOL: Quality of life; T1: Time 1 (the beginning of inpatient rehabilitation); T2: Time 2 (1-month follow-up, at the end of inpatient rehabilitation); CNSD: central nervous system disease; MSD: musculoskeletal system disease; M: mean; SD: standard deviation; df: degrees of freedom; η2: partial eta squared. (DOCX) [file pone.0224503.s003.docx]

|  | CNSD | MSD |  |  |  |
| --- | --- | --- | --- | --- | --- |
| Variable | *M* (*SD*) | *M (SD)* | *F* (*df*) | *p* | *η²* |
| Meaning in life (T1) | 3.43 (0.98) | 3.91 (0.86) | 18.20 (1, 337) | **<.001^a^** | 0.051 |
| Physical QOL (T1) | 2.99 (0.60) | 2.92 (0.47) | 1.32 (1, 337) | .251 | 0.004 |
| Physical QOL (T2) | 3.03 (0.57) | 3.17 (0.56) | 4.05 (1, 337) | **.045^a^** | 0.012 |
| Positive experiences with physical exercises (T1) | 3.03 (0.73) | 3.07 (0.71) | 0.23 (1, 337) | .628 | 0.001 |
| Positive experiences with physical exercises (T2) | 3.06 (0.71) | 3.11 (0.57) | 0.43 (1, 337) | .509 | 0.001 |
| Mobility (T1) | 3.20 (1.03) | 3.50 (0.74) | 8.59 (1, 337) | **.004^a^** | 0.025 |
| Mobility (T2) | 3.26 (0.95) | 3.60 (0.76) | 8.27 (1, 337) | **.004^a^** | 0.035 |
